# Walnut phosphatase 2A proteins interact with basic leucine zipper protein JrVIP1 to regulate osmotic stress response *via* calcium signaling

**DOI:** 10.48130/forres-0024-0012

**Published:** 2024-05-06

**Authors:** Yi He, Shuwen Chen, Chenhao Li, Shen Yang, Zhongyu Sun, Siyu Hou, Zhenggang Xu, Guiyan Yang

**Affiliations:** Shaanxi Province Walnut Engineering Technology Research Center, College of Forestry, Northwest A&F University, Yangling 712100, Shaanxi, China

**Keywords:** *Juglans regia*, Abiotic stress, Expression analysis, Protein interaction, ROS signal

## Abstract

Walnut is an important economic tree species that is susceptible to osmotic stress. Scientific cultivation management is an important way to improve the yield and quality of walnuts, which requires understanding the regulatory mechanisms in response to osmotic stress. Therefore, in this study, 15 protein phosphatase 2A (*PP2A*) genes were identified from the walnut transcriptome (named *JrPP2A01*~*15*) and their potential function responses to osmotic stress were elucidated. The open reading frame (ORF) of *JrPP2A01~15* ranges from 651 to 1,764 bp in length, the molecular weight of the encoded proteins are 24.15−65.61 kDa, and the theoretical isoelectric points are 4.80−8.37. These *JrPP2As* were unevenly distributed on 10 chromosomes and divided into five groups based on the composition of conserved domains, motifs, and exon/intron organizations. The five groups are *JrPP2AAs*, *JrPP2AB's,* *JrPP2AB''s*, *JrPP2AB55s*, and *JrPP2ACs*, including 1, 5, 2, 3, and 4 members, accordingly. The *cis*-elements in *JrPP2As*' promoters were involved in responses to hormone and abiotic stress. Most *JrPP2A* genes, excluding *JrPP2A01*, *JrPP2A02*, *JrPP2A05*, *JrPP2A06*, and *JrPP2A13*, could be induced significantly by PEG_6000_, NaCl, CaCl_2_ and ABA. JrPP2A02, JrPP2A05, JrPP2A07, JrPP2A09, and JrPP2A14, could independently interact with a bZIP transcription factor JrVIP1. Moreover, overexpression of *JrPP2A07, JrPP2A09*, and *JrPP2A14* could significantly decrease ROS accumulation while increasing calcium (Ca) uptake exposed to PEG_6000_ and NaCl stresses, which was mediated by exogenous CaCl_2_ and ABA. These results suggested that *JrPP2A* genes play potential key roles in walnut response to drought and salt-inducing osmotic stress involving Ca- and ABA-dependent signaling pathways.

## Introduction

Walnut is one of the most important economic oil tree species and plays a vital role in rural revitalization and poverty alleviation in China^[1]^. Currently, the planting area of walnut trees ranks first among the four dried fruits (walnuts, almonds, cashews, and hazelnuts). However, the various stresses caused by unstable environmental changes can affect the yield and quality of walnuts, especially drought, abnormal temperature changes, and soil salinization are the main abiotic stimuli restricting the development of the walnut industry in the northwest region of China, which is the main production area. During severe 'late spring cold', all newly sprouted tender branches could be frozen; long-term drought and high temperature can cause different types of diseases and burns, and the commodity rate decreases; saline-alkali soil directly affects the growth and quality of walnuts^[1,2]^. To survive in poor environments, plants have evolved a variety of sophisticated strategies to alleviate damage *via* various pathways, such as releasing stress signals, regulating related genes' expression, physiological responses^[3]^. In regulation mechanism, plant transcription factors (TFs), such as MYB (myeloblastosis), ERF (ethylene-responsive element binding factor), WRKY (containing the WRKYQK protein domain), NAC (NAM/ATAF/CUC domain), bHLH (basic helix-loop-helix), perform an important role in transmitting stress-induced signals and coordinating the functional genes' expression in cells^[4,5]^. In signal transduction networks of developmental processes and stress conditions, plant protein kinases and protein phosphatases play key functions^[6]^. Reactive oxygen species (ROS) signaling is a prominent pathway for plants to respond to stress^[1, 5]^. Protein phosphatase 2A (PP2A), a group of serine/threonine (Ser/Thr) phosphatases, was verified to be involved in the ROS pathway to regulate metabolic changes and cell death^[7]^. Clearly, identification of key factors bound to stress response, such as PP2A, is an important basis for revealing the molecular mechanism of walnut response to osmotic stress.

PP2A exists as a trimer made up of three subunits (A, B and C) in eukaryotic cells. The A, B and C subunits are the structural/scaffold, regulatory, and well-conserved catalytic subunit, accordingly^[8]^. Subunit A is a complex formed by a series of 15 helical HEAT repeats, each repeat consists of approximately 40 leucine residues, which provide a scaffold for the binding of B and C; Subunit C is strongly conserved among different species^[9]^. Subunits A and C shape the core enzyme which interacts with B-subunit to produce the holoenzyme; Subunit B is the 'specificity unit' that determines the specificity of PP2A holoenzyme^[10]^. The B subunit could be further classified into three sub-classes, they are B', B'' and B''' (B55)^[11]^. Genes encoding *PP2A* subunits have been identified and characterized in some plant species. In *Arabidopsis thaliana*, the A, B and C subunit of PP2A contains three, 17 and five members, accordingly^[12]^. In *Hevea brasiliensis*, four, 24 and eight members of A, B and C subunit were identified^[13]^. In other species, the B subunit was also discovered to contain the most members of the three^[8−11]^. There are differences in the quantity of the *PP2A* family among different species, but the B subunit group covers most members, which may be related to the functions of various types of subunits.

PP2A proteins are reported to be involved in several notable biological processes, such as stress-related signaling, which includes abundant hormone-related signal transduction pathways and enzymes^[7−12]^. In *Arabidopsis*, PP2A dephosphorylation and auxin efflux proteins' correct orientation are necessary for auxin polar transport^[14]^; the *PP2A* B subunit B′α and B′β activate brassinosteroid signaling by dephosphorylating the *BZR1* TF in the nucleus^[15]^; the *RCN1*, a *PP2A* A subunit gene, was reported to be involved in the regulation of MeJA/ABA/ethylene signaling^[16]^. In rice, the expression of all catalytic subunit genes (*OsPP2A-1-5*) was significantly up-regulated by salinity stress^[17]^. In wheat, over-expression of *TaPP2AC-1* in transgenic tobacco enhanced drought tolerance through drought-responding signal transduction pathways^[18]^. While the expression of *TaPP2Ac-4B* and *TaPP2Ac-4D* negatively regulated the defense response to *R. cerealis* infection may modulate the expression of certain pathogen-response (PR) and ROS-scavenging-related genes^[19]^. These studies indicated that *PP2A*s have diverse functions in plant stress response, and the walnut *PP2A* family deserves deep attention for revealing the molecular mechanism of stress adaptation.

In recent years, the diverse roles of *PP2A* in herbaceous plants, especially its positive roles in response to some abiotic stresses, have attracted widespread attention. However, little is known about the identification and characterization of *PP2A* genes in woody plants. In this study, we identified the walnut *PP2A* gene family members and further analyzed their phylogenetics, gene structure, chromosome location, conserved motifs, conserved domains, expression patterns, and interaction proteins. Moreover, transgene lines overexpression of candidate walnut *PP2A* genes were obtained to confirm the roles in response to osmotic stress. The results of the current study revealed that walnut *PP2A* genes play positive roles in osmotic stress response by interacting with JrVIP1 protein to control ROS scavenging in a Ca- and ABA-dependent manner.

## Materials and methods

### Plant materials and treatments

Three-year-old 'Xiangling' walnut (a variety widely planted in China) grafting seedlings were planted in flowerpots and grown in a greenhouse at the College of Forestry, Northwest Agriculture and Forestry University (China) with a 14/10 h photoperiod and relative humidity 70% ± 5% under 22 ± 2 °C and used as the materials for the stress treatments. The soil and growth environment as well as the genetic background are consistent. Considering that osmotic stress always resulted from drought, and saline-alkali conditions and *PP2A* genes probably relate to ABA signaling, the plants were treated with 20% (w/v) PEG_6000_, 300 mmol/L NaCl, 20 mmol/L CaCl_2_ and 30 μmol/L ABA by watering the roots, respectively. Normally watered plants were used as controls. After treatment for 5 d, the leaves were collected and stored at −80 °C for RNA isolation. All treatments were replicated three times and each replicate included five seedlings.

### Identification of *PP2A* members from walnut transcriptomes

The walnut leaf transcriptomes under stresses of drought, salt, and ABA (same as above treatments) were sequenced and a preliminary sequence set was obtained. Then firstly 'protein phosphatase' was subjected to search the transcriptomes' database to obtain *PP2A* family candidate sequences, which were further separately blasted (BLAST: Basic Local Alignment Search Tool (nih.gov)) to judge what homologous proteins they belong to. Next, the open reading frame (ORF) of each potential walnut *PP2A* (marked as *JrPP2A*) was confirmed using ORF Finder (www.ncbi.nlm.nih.gov/orffinder). The conserved domains of JrPP2A proteins were confirmed based on the online tools including CD-Search (www.ncbi.nlm.nih.gov/Structure/cdd/wrpsb.cgi), Pfam (http://pfam.janelia.org/), and SMART (http://smart.embl-heidelberg.de/). The conservative motifs were found using MEME online tools (https://meme-suite.org/meme/) and Tbtools^[20]^ with the following parameters: the motif number was 20, any repetition with motif width 17~50. After these analyses, members of the *JrPP2A* family were confirmed. The amino acid number, molecular weight and theoretical isoelectric point (pI) of JrPP2A proteins were analyzed by ExPASy (https://web.expasy.org/protparam/). To clarify the evolutionary relationship of JrPP2A proteins, a neighbor-joining phylogenetic tree with a bootstrap replicate value of 1,000 was constructed in MEGA7 using the JrPP2A proteins, 25 Arabidopsis PP2A proteins downloaded from TAIR and 63 *H. brasiliensis* PP2A proteins downloaded from NCBI (Hevea brasiliensis (ID 503)-Genome-NCBI (nih.gov)). The phylogenetic tree was modified using Evolview (www.evolgenius.info/evolview) and the *JrPP2A* members were grouped referring to the topology of the phylogenetic tree.

### Chromosomal location, gene structure and promoter analysis of *JrPP2A*s

The genomic DNA sequence of *JrPP2A*s were confirmed according to the walnut (*Juglans microcarpa* × *J. regia*) genome (www.ncbi.nlm.nih.gov/genome) and the chromosomal location information of *JrPP2A*s was determined. The exon-intron structure of each *JrPP2A* was defined using Gene Structure Display Server 2.0 (GSDS 2.0: http://gsds.gao-lab.org/). An up-stream 2,000 bp promoter sequence of each *JrPP2A* was obtained according to the genome, and the potential *cis*-acting regulatory elements of the promoters were predicted by PlantCARE (http://bioinformatics.psb.ugent.be/webtools/plantcare/html/).

### Expression analysis of *JrPP2A*s under osmotic stress

The leaves of 3-year-old walnut plants treated separately by PEG_6000_, NaCl, CaCl_2_ and ABA for 5 d were sampled for RNA isolation. The total RNA of each sample was isolated using the cetyltrimethylammonium ammonium bromide (CTAB) method^[1, 5]^ and digested with DNase (Takara, Dalian, China) to ensure the RNA quality. The RNA concentration was tested using the Thermo Scientific™ NanoDrop™ One. 0.5 μg RNA of each sample was reversely transcribed into cDNA using the Prime Script™ RT reagent Kit (CWBIO, Beijing, China). The cDNA was diluted 10-fold with ddH_2_O and used as the template of real-time fluorescent quantitative PCR (qRT-PCR). The 20 μL reaction solution contains 10 μL SYBR Green Real-time PCR Master Mix (CWBIO), 0.5 μM each forward and reverse primer, and 2 μL cDNA template. StepOne™ Real-Time PCR System was adopted to perform the qRT-PCR. The amplification parameters: 94 °C/30 s, followed by 44 cycles of 94 °C/12 s, 60 °C/30 s, 72 °C/40 s, then 81 °C/1 s. The internal reference gene is *18S rRNA* (HE574850)^[21]^. All related primers are listed in Supplemental Table S1. The relative expression levels were calculated using the 2^−ΔΔCᴛ^ method^[22]^.

### Yeast two hybrid assay

Yeast two hybrid (Y2H) assay was used to clarify whether JrPP2A proteins could interact with basic leucine zipper protein (bZIP) JrVIP1. *JrVIP1* was cloned into pGBKT7 vector (marked as BD) to form the bait recombinant (BD-JrVIP1). Each *JrPP2A* was independently inserted into the pGADT7_Rec vector (marked as AD) to generate the prey recombinants (AD-JrPP2As). Meanwhile, *JrVIP1* was inserted into pGADT7_Rec to form AD-JrVIP1, while each *JrPP2A* gene was independently cloned into pGBKT7 to generate BD-JrPP2As. Then the interaction between BD-JrVIP1 and each AD-JrPP2A as well as AD-JrVIP1 and each BD-JrPP2A were confirmed in yeast Y2H by grown on the SD/-Ade/-His/-Leu/-Trp/X-α-Gal/Aureobasidin A (QDO/X/A) medium plates. The empty AD and BD functioned as interaction control^[1,5]^. The related primers were listed in Supplemental Table S2.

### Qualitative and quantitative analysis of reactive oxygen species

To understand the osmotic stress response function, the *JrPP2A* genes were separately inserted into the pROKII vector to generate recombinant vectors 35S::*JrPP2As*. Then each 35S::*JrPP2A* was transformed into *Arabidopsis* using the *Agrobacterium tumefaciens*-mediated method^[1,5]^. The kanamycin-resistant transformed seedlings were further confirmed by PCR and qRT-PCR methods. The most overexpression lines were chosen for osmotic stress response analysis. The seeds of WT and *JrPP2A* transgenic lines were sown on 1/2MS (Murashige and Skoog) agar medium for 12 d, then the seedlings were transfered into soil to grow to one month old for treatments, including 10% PEG_6000_, 10% PEG_6000_ + 10 mol/L CaCl_2_, 10% PEG_6000_ + 10 μmol/L ABA, 100 mmol/L NaCl, 100 mmol/L NaCl + 10 mol/L CaCl_2_, 100 mmol/L NaCl + 10 μmol/L ABA. After 3 d of treatments, the leaves were harvested for reactive oxygen species (ROS) determination. The 3,3'-diaminobenzidine (DAB) and nitroblue tetrazolium (NBT) staining were applied to qualitative confirmation of the ROS generation. The H_2_O_2_ content was determined using the Hydrogen Peroxide Assay Kit (colorimetry, A064-1, NJJCBIO, Nanjing, China). The total ROS content was tested using the chemiluminescence method according to the manufacturer's instructions of the Reactive Oxygen Species Assay Kit (E004, NJJCBIO).

### Determination of calcium content in transgenic Arabidopsis plants

The leaves of WT and *JrPP2A* transgenic lines were ground and dried into a powder, independently. Then each sample powder was placed into a centrifuge tube, in which 1.9 mL of 1% HCl was added and mixed well with a vortex shaker. The mixture was centrifuged (15,000 r/min) after digested at 37 °C for 48 h. 1.5 mL of the supernatant was taken to measure the concentration of Ca^2+^ using a TAS-990 atomic absorption spectrometer at 422.7 nm.

### Pull-down assay

The CDSs of *JrPP2A*s and *JrVIP1* were independently cloned into the vectors of pET30a and pGEX4T-1 and then transformed into Rosetta for expression of JrPP2A-His and GST-JrVIP1 proteins by using 0.1 mM IPTG (isopropyl-b-thiogalactopyranoside). Soluble GST or GST-JrVIP1 fusion proteins were extracted and immobilized using a glutathione HiCap matrix (Qiagen). JrPP2A-His was incubated with immobilized GST or GST-JrVIP1, and the interaction was checked by western blotting analysis. The related primers were included in Supplemental Table S2.

### Statistical analysis

All the data were organized and analyzed using Excel 2023 and SPSS (Chicago, Illinois, USA). The sample variability was described by standard deviation (S.D.) from three repeated assays. The differences between WT and *JrPP2A* transgenic lines were evaluated using Tukey's multiple comparison test (*p* < 0.05).

## Results

### Sequence characteristics and nomination of walnut PP2A proteins

**Figure 1:**
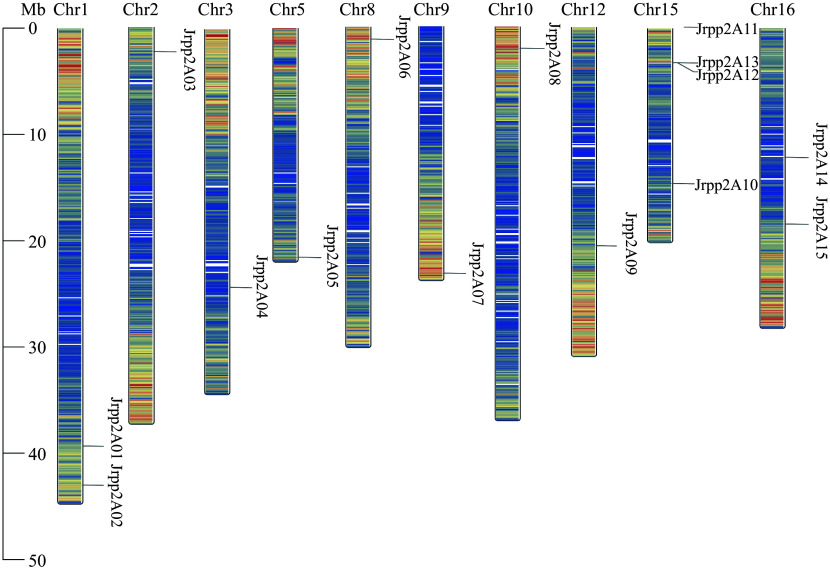
Distribution of the *JrPP2A*s on chromosomes of the *J. regia* genome. The chromosome number is shown on the top side of each chromosome.

A total of 18 putative *JrPP2A* genes were screened from walnut transcriptome, among these 18, three lacked PP2A catalytic domain. Therefore, 15 genes in *J. regia* were identified as *PP2A* family members in the current study. To better describe these 15 *JrPP2A* genes, their positions on the walnut chromosomes were analyzed, and found that these 15 walnut *PP2A* genes were unevenly distributed on 10 chromosomes: Four were distributed on the 15^th^ chromosome, which has the maximum number of *JrPP2A*s, followed by the 01^st^ and 16^th^ chromosome, those have two *JrPP2A*s, accordingly; The chromosomes of 02^nd^, 03^rd^, 05^th^, 09^th^, 10^th^ and 12^th^, each covers only one *JrPP2A* gene. In addition, there was no *JrPP2A* member presented on the chromosomes of the 04^th^, 06^th^, 07^th^, 11^th^, 13^th^ and 14^th^. Considering the conciseness of the description, the *JrPP2A* genes were named *JrPP2A01* to *JrPP2A15* according to their orders in the chromosomes (Fig. 1, Table 1). The ORFs of the 15 *JrPP2A*s were between 651 bp (*JrPP2A12*) and 1,764 bp (*JrPP2A10*), consisting of 216~587 amino acids. The molecular weight of the proteins ranged from 24.15 kDa (JrPP2A12) to 65.61 kDa (JrPP2A10), and the pI ranged from 4.80 (*JrPP2A02*) to 8.37 (*JrPP2A01*) (Table 1).

**Table 1:** Sequence characteristics of 15 *JrPP2A*s.

Gene names	Type	Transcriptome No.	Gene Bank accession No.	Gene ID	Chromosome	ORF length (bp)	Number of amino acids	Molecular weight (kDa)	pI
JrPP2A01	B'	comp30409_c0	XM_018967286.2	LOC108992675	ch1	1,452	483	55.13	8.37
JrPP2A02	B''	comp28147_c0	XM_018971752.2	LOC108996025	ch1	1,254	417	49.44	4.8
JrPP2A03	B55	comp26329_c0	XM_018982199.2	LOC109003870	ch2	1,506	501	56.72	6
JrPP2A04	B'	comp20037_c0	XM_018993415.2	LOC109011996	ch3	1,503	500	57.52	6.24
JrPP2A05	C	comp26715_c0	XM_018952439.2	LOC108981323	ch5	681	226	25.7	4.94
JrPP2A06	B'	comp24655_c0	XM_018979195.2	LOC109001785	ch8	1,503	500	56.94	7.61
JrPP2A07	C	comp32898_c0	XM_018969187.2	LOC108994090	ch9	918	305	34.78	5.24
JrPP2A08	B'	comp25187_c0	XM_018958784.2	LOC108986228	ch10	1,575	524	59.87	7.97
JrPP2A09	B''	comp9850_c0	XM_018985083.2	LOC109005962	ch12	1,431	476	54.74	4.93
JrPP2A10	A	comp28348_c0	XM_035685846.1	LOC109013629	ch15	1,764	587	65.61	4.88
JrPP2A11	B55	comp28413_c1	XM_018989098.2	LOC109008856	ch15	918	305	34.06	5.16
JrPP2A12	B55	comp28413_c1	XM_018989097.2	LOC109008856	ch15	651	216	24.15	6.21
JrPP2A13	C	comp27670_c1	XM_018980847.2	LOC109002920	ch15	912	303	34.84	4.88
JrPP2A14	C	comp23892_c0	XM_018963562.2	LOC108989812	ch16	921	306	35.01	4.83
JrPP2A15	B'	comp13497_c0	XM_018951013.2	LOC108980163	ch16	1,530	509	58.52	7.18

### Classification of JrPP2A proteins according to phylogenesis and gene structure

**Figure 2:**
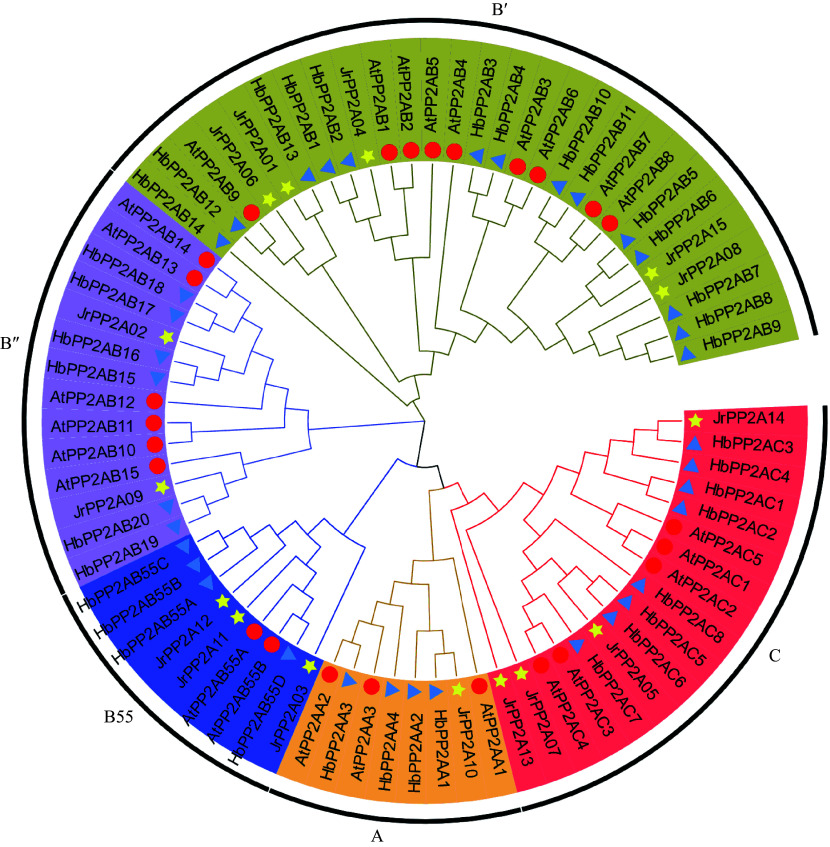
Phylogenetic relationship of PP2A proteins from *J. regia*, *A. thaliana* and *H. brasiliensis*. A, C, B55, B', B" means five sub-family of *PP2A*s, respectively, which are displayed in different colors. A total of 15 walnut PP2As are represented by yellow five-pointed stars, 25 Arabidopsis PP2As are represented by red circles, 36 *H. brasiliensis* PP2As are represented by blue triangles.

Classification may have a potential relationship with gene function. Therefore, the 15 JrPP2A proteins were classified according to the widely accepted approaches mainly including evolutionary relationship and gene structure. To investigate the genetic relationship, total 25 Arabidopsis *PP2A*s (three A subunits-*AtPP2AA*, nine B' subunits-*AtPP2AB'*, six B'' subunits-*AtPP2AB''*, two B55 subunits-*AtPP2AB55*, and five C subunits-*AtPP2AC*), 36 rubber tree *PP2A*s (four A subunits-*HbPP2AA*, 14 B' subunits-*HbPP2AB'*, six B'' subunits-*HbPP2AB"*, four B55 subunits-*HbPP2AB55*, and eight C subunits-*HbPP2AC*) as well as the 15 walnut *PP2A*s were aligned to construct a phylogenetic tree using NJ method. As shown in Fig. 2, these PP2A proteins were divided into five clusters according to the branch of the evolutionary tree and subunits covered in *Arabidopsis* and rubber PP2As. The B' cluster covered most PP2A proteins, including five JrPP2As (JrPP2A01, JrPP2A04, JrPP2A06, JrPP2A08, JrPP2A15), nine AtPP2As and fourteen HbPP2As. Group A had the least number of PP2As, containing one JrPP2A (JrPP2A10), three AtPP2As and four HbPP2As. Subfamily B'' covered 2 JrPP2A proteins (JrPP202, JrPP209), while subclass B55 included three JrPP2As (JrPP203, JrPP211, JrPP212). The other four JrPP2As (JrPP2A05, JrPP2A07, JrPP2A13, JrPP2A14) were clustered in C subgroup (Fig. 2).

**Figure 3:**
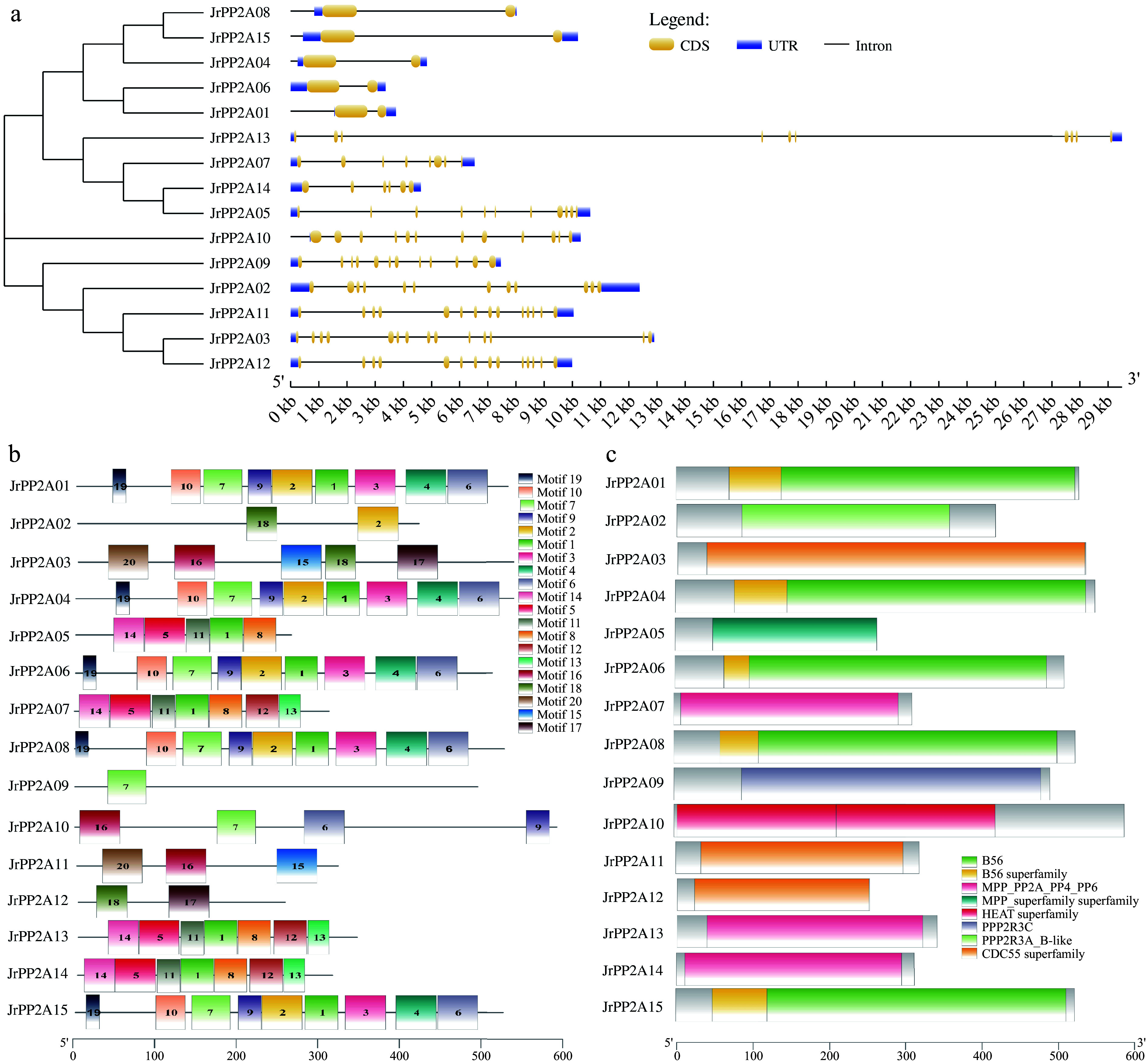
Gene structure of walnut *PP2A*s. (a) Exon-intron distribution map. The vertical phylogenetic tree and gene structure of *JrPP2A*s was constructed by GSDS online software. Yellow boxes indicate exons; blue boxes indicate upstream or downstream; black lines indicate introns. (b) Conserved motif analysis: 20 separate patterns were identified with the MEME suite and each pattern was depicted with different colors. (c) Distribution of conserved domains.

Considering that the exon-intron structure can provide prominent evidence supporting the phylogenetic relationships of a gene family, the diversity of the intron-exon construction leads to the gene structure being varied. Thus, a structural analysis was performed using these 15 CDSs and genomic sequences of *JrPP2A* genes in the GSDS online. As shown in Fig. 3a, the intron numbers of the 15 *JrPP2A* genes varied widely among the five subfamilies. In detail, the A and B" sub-family *JrPP2A* genes contained 12 and 11 introns, accordingly. The intron numbers of C subclass *JrPP2A* genes ranged from 6 (*JrPP2A14*) to 10 (*JrPP2A13*). The B55-like *JrPP2A* genes possessed the most introns (14), while the B' sub-group *JrPP2A* genes had the fewest introns (only 1~2). The performance of the introns validated that the structure of exon–intron was similar in the same subfamily, despite some differences in the length of exons. The showing exon–intron structure was consistent with the evolutionary relationship.

### Conserved motifs and domains of walnut PP2A proteins

A total of 20 conserved motifs were identified from the 15 walnut PP2A proteins using an online MEME tool and the basic information (width and best possible match sequence) was downloaded and displayed in Table 2. Each motif included 17–50 amino acids. The proteins categorized within the same group shared a similar motif composition, which further verified the group definitions. As shown in Fig. 3b, some unique motifs could be detected only in specific subgroups. In detail, motif 19, motif 10, motif 7, motif 9, motif 2, motif 1, motif 3, motif 4 and motif 6 was detected in B' subunit JrPP2A proteins; Motif 14, motif 5, motif 11, motif 1 and motif 8 was found in all C subunit JrPP2As. Motif 16, motif 7, motif 6 and motif 9 was contained in A subunit of JrPP2As; Motif 20, motif 6, motif 15, motif 18 and motif 17 was located in subgroup B55 JrPP2As; while subgroup B'' JrPP2A proteins only contained motif18, motif2 and motif7.

**Table 2:** Motif sequences identified by the MEME tool.

Motif	Width	Motif consensus
1	41	TIVYGFYDETERHNGIAELLEIFGSIIDGFALPLKEEHKIF
2	50	KVAKRYIDHSFVLRLLDLFDSEDPREREYLKTILHRIYGKFMVHRPFIRK
3	50	HKPKSIGLYHQQLSYCITQFVEKDPKLADTVIRGLLKYWPVTNSQKEVMF
4	50	PAEFQRCMVPLFRQIGCCLNSSHFQVAERALFLWNNDHIVNLIAQNRNVI
5	50	NVQPVKSPVTICGDIHGQFHDLIELFRIGGNCPDTNYLFMGDYVDRGYYS
6	50	PIIFPALEKNARSHWNQAVQNLTLNVRKIFSEMDPELFEECQRQFQEDEA
7	48	DIKRQTLIELVDFVASGSGKFTETAIQEMIKMVSVNLFRVLPPKPREN
8	41	CLHGGLSPSIETLDNIRVIDRIQEVPHEGPMCDLLWSDPDD
9	29	EPSFDPAWPHLQJVYELLLRFVSSSETDA
10	37	VEALPAFKDVPNSEKQNLFISKLNLCCVVFDFSDPTK
11	29	ETFTLLLALKVRYPDRITJLRGNHESRQI
12	41	WGVSPRGAGYLFGGDVVSQFNHTNNLDLICRAHQLVMEGYK
13	27	WFQDKGIVTVWSAPNYCYRCGNVAAIL
14	38	SHADLDRQIEQLKECKPLPEAEVKVLCDKAKEILVEES
15	50	AHAHDFNINSISNNSDGETFISADDLRINLWNLEISNQCFNIIDMKPANM
16	50	YKTEFQSHEPEFDYLKSLEIEEKINKIRWCQTQNGALFLLSSNDKTIKFW
17	50	MDSGPVATFKVHENLRPKLCELYENDSIFDKFECCJSGDGJHFATGSYSN
18	38	EVITSAEFHPIHCNLLAYSSSRGFIRLIDMRCSALCDQ
19	17	TMIKQILSKLPRKPSKS
20	50	PLEWKFSQVFGERPAGEEVQEVDIISAIEFDKSGDHLAVGDRGGRVVJFE

The conserved domains of JrPP2A proteins further confirmed the sub-classifications that were presented by the motifs. As shown in Fig. 3c, the different subunits of JrPP2As were obviously varied, the A sub-family members covered the 'HEAT superfamily' domain, the B sub-family members covered the 'B56 superfamily' domain, the C sub-family members covered the 'MPP superfamily' domain, the B55 sub-family members covered 'CDC55 superfamily' domain, the B'' sub-family members covered 'PPP2R superfamily' domain.

### The* cis*-acting regulatory elements in *JrPP2A*s' promoters

To analyze the potential transcriptional regulation of *JrPP2A* genes in abiotic stress response, putative *cis*-acting elements in *JrPP2A* promoters were identified. As a result, a total of 55 *cis*-acting elements in the promoters were identified and the *JrPP2A* genes in the same subfamily possessed similar *cis*-elements. These elements belonged to four types: hormone responsiveness, light responsiveness, abiotic stress response, plant growth, and development (Supplemental Table S3). The elements in 'abiotic stress response' and 'light-responsive' were abundant with 15 and 22 hits (Supplemental Tables S4 & S5), accordingly. The elements Box 4 and G box (light responsive related), MYC (drought and ABA responsiveness related), and MYB (drought-inducible) could be found in most *JrPP2As*' promoters. In addition, 70% of the *JrPP2A* genes covered ABA-responsive element (ABRE), suggesting that *JrPP2A*s may be associated with the regulation of the ABA pathway. Meanwhile, some elements such as CAT-box and O2 were predicted to be involved in plant growth and development (Supplemental Tables S3−S5). These results implied that the walnut *PP2A* genes may play abundant functions in plant growth, development and response to abiotic stresses.

### Expression patterns of *JrPP2A* genes under osmotic stresses and ABA

To validate the possible functions of *JrPP2A* genes in osmotic stress response and whether involving in ABA signaling, the transcript abundance of 15 selected *JrPP2A* genes were analyzed under drought (PEG_6000_), salt (NaCl), calcium (CaCl_2_) and ABA treatment.

#### Under PEG_6000_ treatment

**Figure 4:**
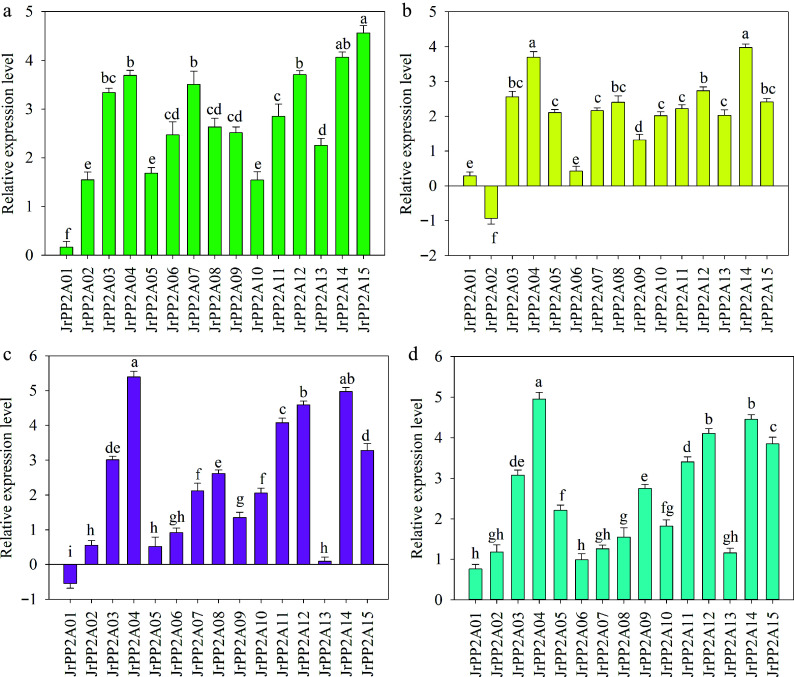
QRT-PCR analysis of the expression of *JrPP2A* genes under drought, NaCl, CaCl_2, _and ABA treatments. The relative expression level is expressed as relative to the internal reference gene and at 0 h. Error bars represent the SD (n = 3). Lowercase letters indicate significant differences among different *JrPP2A* genes under each treatment according to the Student's t-test (*p* < 0.05). (a) PEG_6000_ stress. (b) NaCl stress. (c) CaCl_2_ treatment. (d) ABA treatment.

The response of *JrPP2A* genes to drought stress was different. *JrPP2A15* was up-regulated to the highest level while *JrPP2A01* was the lowest one. *JrPP2A15* was induced to 1.12-~27.86-fold of other genes. *JrPP2A14* was also up-regulated to exceed 4.00 and ranked just after *JrPP2A15*. The expression value of *JrPP2A12*, *JrPP2A04,* and *JrPP2A03* exceeded 3.00, while the transcription level of *JrPP2A02*, *JrPP2A0*5, and *JrPP2A10* was 1.54~1.68. The other five (*JrPP2A06*, *JrPP2A08*, *JrPP2A09*, *JrPP2A11*, *JrPP2A13*) genes differed not obviously with a level between 2.26 and 2.85 (Fig. 4a).

#### Response to NaCl stress

Most of the *JrPP2A* genes except *JrPP2A02* were induced by NaCl with varied levels. The expression level of *JrPP2A14* was the maximum while not significantly different from the second gene *JrPP2A04*. The transcription values of *JrPP2A12*, *JrPP2A03*, *JrPP2A08,* and *JrPP2A15* were similar and ranged from 2.74 to 2.42. The expression levels of *JrPP2A05*, *JrPP2A07*, *JrPP2A10*, *JrPP2A11,* and *JrPP2A13* were changed not significantly, their values were 2.01~2.22. The expression value of *JrPP2A09* (1.32) was significantly lower than the above 10 up-regulated genes while higher than the other three (*JrPP2A01*, *JrPP2A02,* and *JrPP2A06*). Meanwhile, *JrPP2A01* and *JrPP2A06* displayed similar expression levels (Fig. 4b).

#### Under CaCl_2_ treatment

Most *JrPP2A* genes excluding *JrPP2A01* were induced by CaCl_2_ with a large range (0.55~5.39). The expressions of *JrPP2A02*, *JrPP2A05*, *JrPP2A06,* and *JrPP2A13* were at a level less than 1.00. *JrPP2A09*, *JrPP2A07*, *JrPP2A08,* and *JrPP2A10* were up-regulated to 2.06~2.62, in which *JrPP2A08* was significantly higher than *JrPP2A07* and *JrPP2A10*. *JrPP2A03* and *JrPP2A15* were similar with the expression values 3.01 and 3.28, accordingly. Meanwhile, the difference between *JrPP2A03* and *JrPP2A08* was not significant. The other four, *JrPP2A04*, *JrPP2A11*, *JrPP2A12,* and *JrPP2A14*, was showed most obviously transcription and exceed 4.00 (Fig. 4c).

#### Under ABA treatment

All *JrPP2A* genes were induced by ABA. *JrPP2A04* was transcribed highest and significantly differed from all others. The expression levels of *JrPP2A14* and *JrPP2A12* also exceeded 4.00 and ranked second and third, accordingly, with a non-significant difference. *JrPP2A15*, *JrPP2A11* and *JrPP2A03* were up-regulated to a level ranging from 3 to 4. The expression values of *JrPP2A09* and *JrPP2A05* were 2.22~2.75 with significant difference. *JrPP2A01* and *JrPP2A06* were transcribed with a level lower than 1.00. The other five genes (*JrPP2A02*, *JrPP2A07*, *JrPP2A08*, *JrPP2A10* and *JrPP2A13*) were expressed in the level between 1.16~1.82 (Fig. 4d).

### Overexpression of *JrPP2A*s promote osmotic stress tolerance involving in Ca and ABA signal

**Figure 5:**
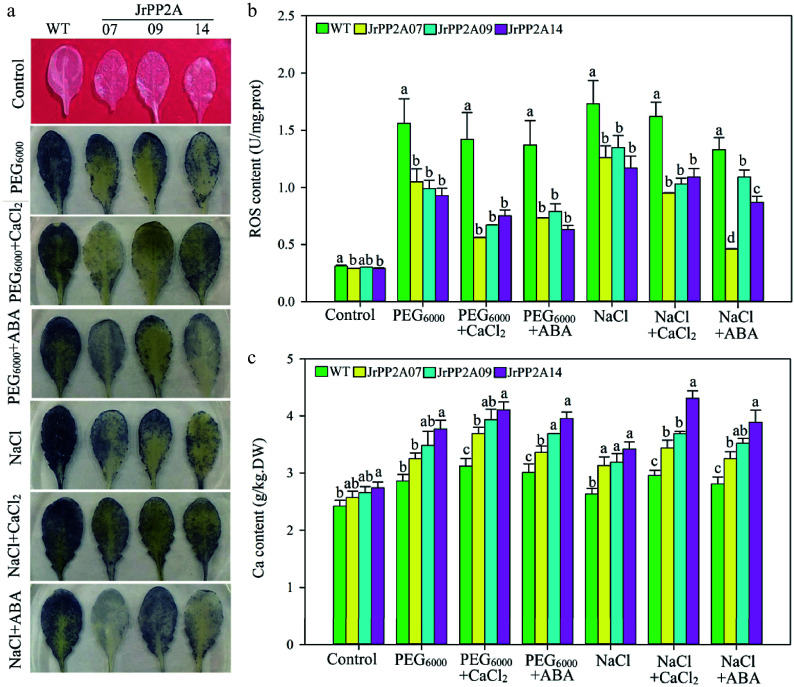
Osmotic stress response function of *JrPP2A*s. WT, wild type. JrPP2A07, 09, 14, the transgenic lines overexpression of *JrPP2A07*, *JrPP2A09*, *JrPP2A14*. Fourty two-day old seedlings were treated with PEG_6000_, PEG_6000 _+ CaCl_2_, PEG_6000 _+ ABA, NaCl, NaCl + CaCl_2_, NaCl + ABA for 5 d. Control was normally watered. The significant differences among WT, *JrPP2A07*, *JrPP2A09,* and *JrPP2A14* were marked with lowercase (*p* < 0.05). (a) NBT staining. (b) Total ROS content. (c) Total Ca content.

To confirm the roles of *JrPP2A*s in osmotic stress response, three members, *JrPP2A07*, *JrPP2A9, *and *JrPP2A14*, were chosen according to classification and expression under PEG_6000_, NaCl, CaCl_2_, and ABA treatments. *JrPP2A07*, *JrPP2A9,* and *JrPP2A14* were separately overexpressed in *A. thaliana*. The transgenic lines with the highest expression value (160.89-, 112.20-, 101.83-fold of WT, accordingly) were selected for analysis (Supplemental Fig. S1a). The results showed that the 42-d old of WT and transgenic seedlings were grown normally without ROS generation under control condition, however, when exposed to PEG_6000_ and NaCl stress, NBT straining (represent O^2−^) of WT was deeper than that of transgenic lines (Fig. 5a). Quantitative determination of H_2_O_2_ also revealed a similar pattern as NBT staining (Supplemental Fig. S2a). The total ROS content of WT was 1.49-~1.68-fold and 1.37-~1.48-fold of *JrPP2A07*, *JrPP2A9*, and *JrPP2A14* transgenic lines under PEG_6000_ and NaCl stress, accordingly (Fig. 5b). Moreover, the cell damage reflected by electrolyte leakage (EL) rate was similar to ROS accumulation. The EL rate of WT was 1.53-~1.91-fold and 1.57-~1.73-fold of *JrPP2A07*, *JrPP2A9*, and *JrPP2A14* transgenic lines under PEG_6000_ and NaCl stress, accordingly (Supplemental Fig. S1b). However, the changes of catalase (CAT) activity were opposite to that of ROS content and EL rate (Supplemental Fig. S1c). These results suggested that *JrPP2A07*, *JrPP2A9*, *JrPP2A14* could positively improve plant drought and salt inducing osmotic stress tolerance.

Considering the up-regulating expression by CaCl_2_ and ABA, we want to know whether the *JrPP2A* genes are associated with Ca and ABA signaling when responding to osmotic stress. Therefore, the 42-day old seedlings were also treated simultaneously with PEG_6000_ and CaCl_2_, PEG_6000_ and ABA, NaCl and CaCl_2_, NaCl and ABA. Interestingly, we observed that compared to pure PEG_6000_ and NaCl treatments, the addition of CaCl_2_ and ABA resulted in changes in resistance physiological indicators of *JrPP2A*s' transgenic plants, suggesting further improvement in osmotic stress resistance (Fig. 5a & b, Supplemental Fig. S1b & S1c). The NBT staining, H_2_O_2_ content and total ROS accumulation under PEG_6000 _+ CaCl_2_ and PEG_6000_ + ABA was less than under PEG_6000_, under NaCl + CaCl_2_ and NaCl + ABA was less than under NaCl, accordingly (Fig. 5a & b, Supplemental Fig. S2a). The cell damage was alleviated after adding CdCl_2_ and ABA. The EL rate of transgenic lines under NaCl + ABA was 52.56%~84.00% of that under NaCl (Supplemental Fig. S1b), while the CAT activity was increased by adding CdCl_2_ and ABA (Supplemental Fig. S1c). Moreover, the Ca uptake in *JrPP2A* transgenic plant cells was improved compared to WT. Under control conditions, the Ca content in transgenic plants' cells was 1.06-~1.13-fold of WT. When exposed to PEG_6000_ and NaCl, the degree of Ca increases in transgenic plants was also higher than that of WT. The Ca content of WT and transgenic lines under PEG_6000_ stress were 1.18-~1.38-fold of themselves under control. Adding CaCl_2_ and ABA significantly improved the Ca accumulation compared to a single treatment of PEG_6000_ and NaCl (Fig.5c), further confirmation of the relationship of Ca with osmotic stress in *JrPP2A* genes' response pathway.

### Interactions between JrVIP1 protein and JrPP2As

**Figure 6:**
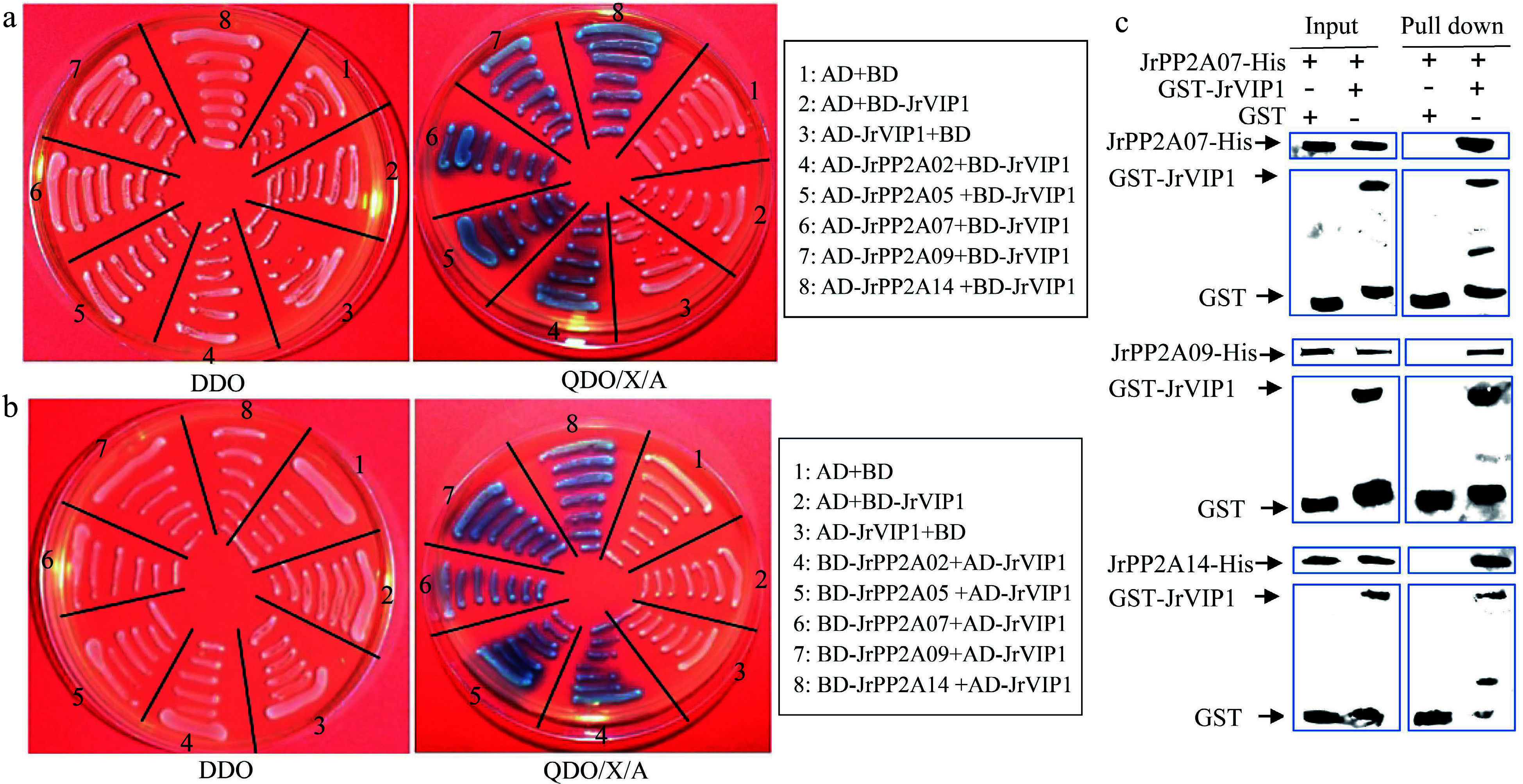
Interaction analysis of JrVIP1 and JrPP2A proteins using yeast two-hybrid (Y2H) and *in vitro* pull-down assay. AD+BD, AD+BD-JrVIP1, AD-JrVIP1+BD, negative control. The DDO plate was used as positive control for growth. (a) JrVIP1 was used as the bait. (b) JrVIP1 was used as the pray. (c) An *in vitro* pull-down assay demonstrates the interaction between JrVIP1 with JrPP2A07, JrPP2A09, JrPP2A14. JrPP2A-His protein was incubated with immobilized GST or GST-JrVIP1 protein, and immunoprecipitated fractions were detected by anti-His antibody. The assay was performed three times with the same result.

JrVIP1 is a basic leucine zipper protein (bZIP) and homologous to the VirE2-INTERACTING PROTEIN1 (VIP1) from *A. thaliana* (Supplemental Fig. S3), whose dephosphorylation can be mediated by PP2A in osmotic and other stresses^[11, 23]^. In our previous study, to analyze the relationship between walnut response to drought and demethylation, we constructed BD-JrVIP1, which was used to screen the possible interacting proteins from the walnut yeast two hybrid library, and found that there were many JrPP2A members. To very whether the biological function of the *JrPP2A* family members was associated with *JrVIP1*, in this study, AD-JrPP2As were constructed and submitted to the Y2H system to confirm the interaction with BD-JrVIP1. The results showed that JrPP2A02, JrPP2A05, JrPP2A07, JrPP2A09, and JrPP2A14 could interact with JrVIP1, respectively (Fig. 6a). The interactions were also tested by AD-JrVIP1 and BD-JrPP2As (Fig. 6b). Moreover, JrPP2A07, JrPP2A09, and JrPP2A14 were selected to further verify their interactions with JrVIP1 by an *in vitro* pull-down assay (Fig. 6c). These data confirmed that JrVIP1 could interact with JrPP2A07, JrPP2A09, and JrPP2A14. Since VIP1 homologous were believed to be a vital protein for phosphorylation or dephosphorylation in stress response^[11, 23]^, we are more convinced that the *JrPP2A* genes are related to stress response involving in phosphorylation.

## Discussion

The plant *PP2A* gene has been regarded as important members in response to various external stimulus^[24, 25]^. To provide scientific guidance in the cultivation and management of walnut trees and then to guarantee the yield and quality of walnuts under adverse conditions, the molecular mechanism of adaptation to adversity is urgently to be revealed. Many environmental factors may lead to osmotic stress, so the regulation of osmotic stress response is particularly noteworthy. Therefore, the walnut transcriptomes under different abiotic stresses were sequenced to establish a basic database for identifying key genes that adapt or respond to environmental stresses. In the current study, 15 *JrPP2A* genes with prominent expression levels that can respond to various stressors were identified from the transcriptome data mentioned above. This quantity, 15 *JrPP2A* genes, is less than the number of members of the *PP2A* family in other plants such as *Arabidopsis* and rubber trees. The possible reason is that the *JrPP2A* family genes selected in this study are shared genes screened from transcriptomes under various stresses, and mainly transcriptionally expressed genes that respond to osmotic stress. It cannot be guaranteed that all members can be induced to express under stress, nor can these genes be screened in the transcriptome database. Walnuts are likely to have other *PP2A*s that may not be clear about their response to osmotic stress. In terms of evolutionary relationship, the 15 JrPP2A proteins shared a high similarity with the *PP2A*s from other species, such as *A. thaliana*^[11, 23]^, *O. sativ*^[26]^, and *H. brasiliensis*^[13]^ and grouped into five subfamilies (Fig. 1, Table 1). The ORF length, pI, amino acid number, and molecular weight of *JrPP2A*s were similar to those from Arabidopsis and *H. brasiliensis*^[11,13,27]^, confirming that these 15 *JrPP2A*s belong to PP2A protein family.

To understand the biological functions of *PP2A* family members, originally, both intron/exon organizations and protein motif patterns were analyzed, and found that *JrPP2A*s displayed diverse gene and protein structures. The intron/exon structure is an important pathway for gene functional evolution and the main reason for functional differences with its homologous proteins^[28]^. Despite the conserved distribution pattern of exons in subgroups B and C, many other subgroups displayed diversity in exon structure and number, which was consistent with the protein pattern (Fig. 3). Most of B' subunit type JrPP2As contained motifs of 19, 10, 7, 9, 2, 1, 3, 4, and 6, which were related to B56 domain^[7, 13, 24]^; while JrPP2ACs were related to MPP domain^[7, 24]^ (Fig. 3c). Because different motifs and the number of motifs were related to functions^[25−27]^, our results suggested tha*t JrPP2A*s have potential abundant roles in walnut. Next, given that plant promoter *cis*-acting elements perform essential roles in regulating gene expression and may imply metabolic pathways and stress response^[29,30]^, the *cis*-acting elements in *JrPP2A*s promoters were detected. We screened abundant *cis*-elements related to drought and salt stress response (Supplemental Tables S3−S5), such as MYC elements, W-box, MBS elements, MYB recognition site, TCA-element. The results preliminarily suggested that these *JrPP2A*s may be involved in stress responses such as drought and salt regulated by the upstream promoter elements.

Consequently, the response patterns of 15 *JrPP2A* genes exposed to PEG_6000_ and NaCl were determined and found that most of the *JrPP2A*s were significantly induced by PEG_6000_ and NaCl. The transcription activity of genes in response to different stresses may effectively predict their potential functions. *Arabidopsis* *PP2AC5* was reported to be induced by drought and salt stresses, when it was overexpressed, the transgenic *Arabidopsis* was verified to improve the drought and salt stress tolerance^[31]^. Compared with the loss-of-function mutant *pp2a-c5-1,* *PP2AC5* overexpression lines were conferred with better root and shoot growth under salt treatments^[32]^. The drought and salt inducible *GmPP2A-B"71* could also enhance plant tolerance to drought and salt stresses via overexpression in soybean^[33]^. A novel Ca^2+^-binding protein, named AtCP1 (AtPP2A-B"43), can be up-regulated by NaCl treatment, was also believed to positive on slat stress response^[34]^. The mRNA levels of *Solanum tuberosum* *PP2Ac1*, *PP2Ac2a*, *PP2Ac2b* and *PP2Ac3* in leaves were up-regulated by salt stress, suggesting that the subunits might have vital roles in response to drought and salt stress^[35]^. These reports and the inducible expression of *JrPP2A*s let us believe that *JrPP2A* genes are likely to play roles in drought and salt stress response. From the expression level, *JrPP2A04*, *JrPP2A14* and *JrPP2A15* were the top three while *JrPP2A01* and *JrPP2A02* were the bottom two those may deserve further attention (Fig. 4a−c).

To confirm the functions of *JrPP2A* genes in drought and salt-inducing osmotic stress response, *JrPP2A07*, *JrPP2A09*, and *JrPP2A14* were independently overexpressed in *A. thaliana* and found that the transgenic plants showed less ROS accumulation, and lighter cell damage while higher antioxidant enzyme activity than those of WT under PEG_6000_ and NaCl stress (Supplemental Figs S1 & S2a, Fig. 5), determining that *JrPP2A07*, *JrPP2A09*, and *JrPP2A14* are positive genes in walnut osmotic stress tolerance. Additionally, the process that plant *PP2A* responding to osmotic stress is implicated in ABA signaling^[36]^. In this study, the transcription of *JrPP2A*s under ABA treatment was tested and showed that B subunit *JrPP2A* genes were induced obviously by ABA, especially *JrPP2A04*. In other species, such as wheat, *TaPP2AB-α*, a novel B subunit of *PP2A*, was induced by the response to NaCl, PEG_6000_, cold, and ABA at the transcriptional level^[37]^. Transgenic Arabidopsis overexpression of *TaPP2AB-α* displayed more lateral roots under mannitol or NaCl treatment^[37]^. These results indicated that *TaPP2AB-α* could promote plant lateral root growth under osmotic conditions^[37]^. TIP41, an interactor of PP2A present in *Arabidopsis*, was induced by long-term NaCl, polyethylene glycol and ABA treatments, proving that *TIP41* mediates the participation of *PP2A* in ABA-mediated mechanisms^[38]^. The mRNA levels of *A. thaliana* *PP2AA3* in roots and shoots were up and down-regulated by drought and ABA treatments, suggesting that the subunits might have vital roles in response to drought and ABA treatments^[39]^. Therefore, we believed that *JrPP2A*s in response to osmotic stress involving in ABA signaling.

**Figure 7:**
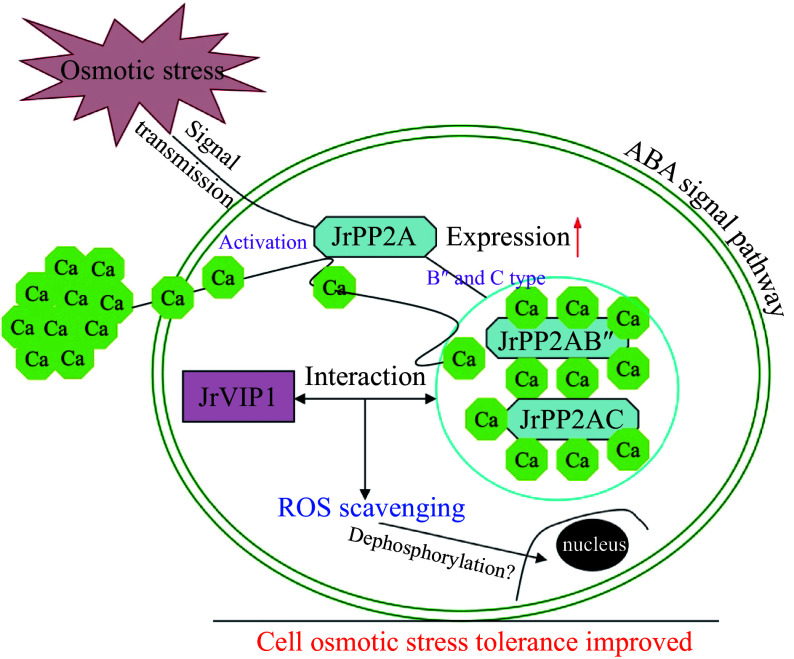
The *JrVIP1* and *JrPP2A*s mediated osmotic stress responsive pathway in walnut trees.

Under external stimulation, Arabidopsis PP2A protein stimulates the Ca accumulation or transport within cells and in turn activates B'' and C class PP2A proteins^[11, 23]^. To investigate whether the *JrPP2A* family genes are involved in calcium signaling in osmotic stress response, we also invested the transcription activity of the *JrPP2A* genes under the condition of adding exogenous CaCl_2_. We were surprised to find that members of *JrPP2A* genes were significantly induced by CdCl_2_ (Fig. 4d). Overexpression of *JrPP2A07*, *JrPP2A09*, and *JrPP2A14* in Arabidopsis promoted the Ca accumulation within plant cells under PEG_6000_ and NaCl stress. Moreover, under CaCl_2_ mediation conditions, the ROS accumulation and cell damage were reduced, while antioxidant protection was enhanced, indicating the effective improvement of the plant osmotic stress tolerance with increasing Ca uptake (Supplemental Figs S1 & S2a, Fig. 5). These performances confirmed the positive mediation of Ca in *JrPP2A*s' abiotic stress response. The activation of B'' and C subunits of PP2A proteins mediates the dephosphorylation of VIP1^[11, 23]^. Hypo-osmotic stress regulates the dephosphorylation and nuclear-localization of VIP1. When cells confronted with mechanical stress, *VIP1* was transiently accumulated in the nucleus, where it controls the expression of the target genes and inhibits mechanical stress-induced root waving^[11]^. In this process, Ca signal was accompanied to play necessary roles^[23]^. These reports told that the interaction between PP2A and VIP is an important pathway for osmotic stress response. In walnut tree, we also discovered that JrVIP1, a homology of Arabidopsis VIP1, could interact with B'' subunit type JrPP2A02 and JrPP2A09 as well as C subunit type JrPP2A05, JrPP2A07 and JrPP2A14 (Fig. 6). Moreover, *JrVIP1* was also up-regulated by osmotic stress (Supplemental Fig. S2b & S2c). Therefore, we believe that the *JrPP2A* genes also mediate VIP1 dephosphorylation in response to osmotic stress and are involved in Ca signaling. Meanwhile, an osmotic stress responsive pathway mediated by JrVIP1 and JrPP2As was summarized as Fig. 7.

## Conclusions

In this study, we identified the *PP2A* family genes from the walnut transcriptome and a total of 15 *JrPP2A*s were screened to be unevenly distributed on 10 of the walnut chromosomes. The *JrPP2A*s genes were grouped into five subfamilies and members of the same subfamily shared similar gene structures and conserved protein motifs. Promoter element compositions imply that *JrPP2A*s may be involved in hormone, light, growth, development, and abiotic stress responses. Most of the *JrPP2A* genes exhibited various expression levels to drought and salt-inducing osmotic stress, among which *JrPP2A07*, *JrPP2A09*, and *JrPP2A14* were improved to play positive roles in osmotic stress response involving Ca and ABA signaling. Moreover, B'' and C types of JrPP2A proteins mediate the dephosphorylation of JrVIP1 in osmotic stress response. Our findings generate novel insights into *PP2A* family genes in walnut and lay a foundation for further understanding their biological functions.

## SUPPLEMENTARY DATA

Supplementary data to this article can be found online.

## Data Availability

All the data were presented in the main manuscript and additional supporting files. The Arabidopsis and *H. brasiliensis* related datasets generated and/or analyzed during the current study are available in the TAIR database (www.arabidopsis.org) and NCBI (*Hevea brasiliensis* (ID 503)-Genome-NCBI (nih.gov)).
