# Supplementary material for: Walnut phosphatase 2A proteins interact with basic leucine zipper protein JrVIP1 to regulate osmotic stress response via calcium signaling
Source: For Res (Fayettev). 2024 May 6;4:e016. doi: 10.48130/forres-0024-0012 (PMC11543299; doi:10.48130/forres-0024-0012)
Supplement: Supplementary file 1 — Supplementary data to this article can be found online. [file forres-0024-0012-S1.zip › 10.48130_forres-0024-0012-Suppl-FigureS2.pdf]

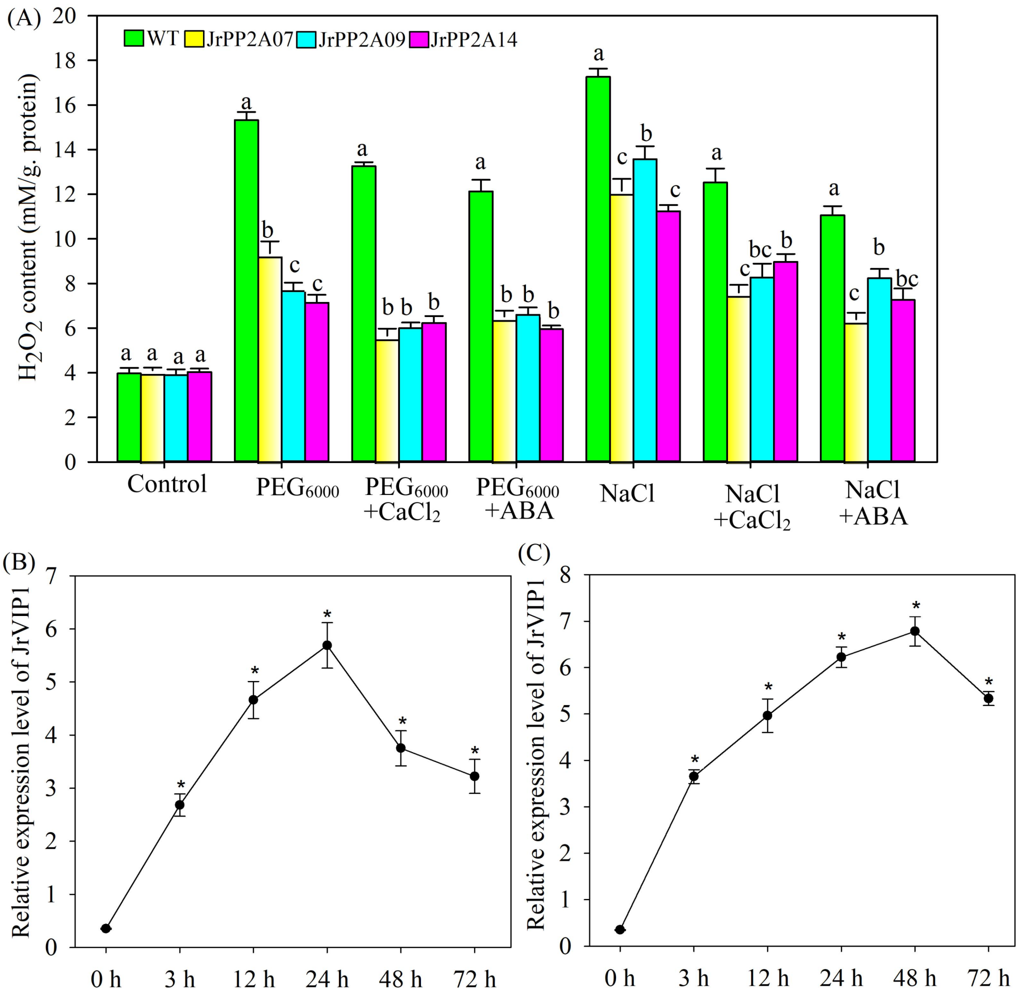

Fig. S2 The generated H_2O_2 contents of *JrPP2A* transgenic plants under osmotic stress same as Fig. 5 (A) and the expression patterns of *JrVIP1* under PEG_{6000} (B) as well as NaCl (C) stress. The significant differences among WT, *JrPP2A07*, *JrPP2A09* and *JrPP2A14* were marked with lowercase (P<0.05) in A. * in B and C means the differences between the treatment time points and 0 h (control) was significant (P<0.05). For the stresses, 20% (w/v) PEG_{6000} and 0.3 mol/L NaCl was independently watered to the 3-year-old 'Xiangling' walnut for 3 days, and the leaves were sampled at 0 (Control), 3, 12, 24, 48, 72 h.
