# Supplementary material for: Walnut phosphatase 2A proteins interact with basic leucine zipper protein JrVIP1 to regulate osmotic stress response via calcium signaling
Source: For Res (Fayettev). 2024 May 6;4:e016. doi: 10.48130/forres-0024-0012 (PMC11543299; doi:10.48130/forres-0024-0012)
Supplement: Supplementary file 1 — Supplementary data to this article can be found online. [file forres-0024-0012-S1.zip › 10.48130_forres-0024-0012-Suppl-FigureS3.pdf]

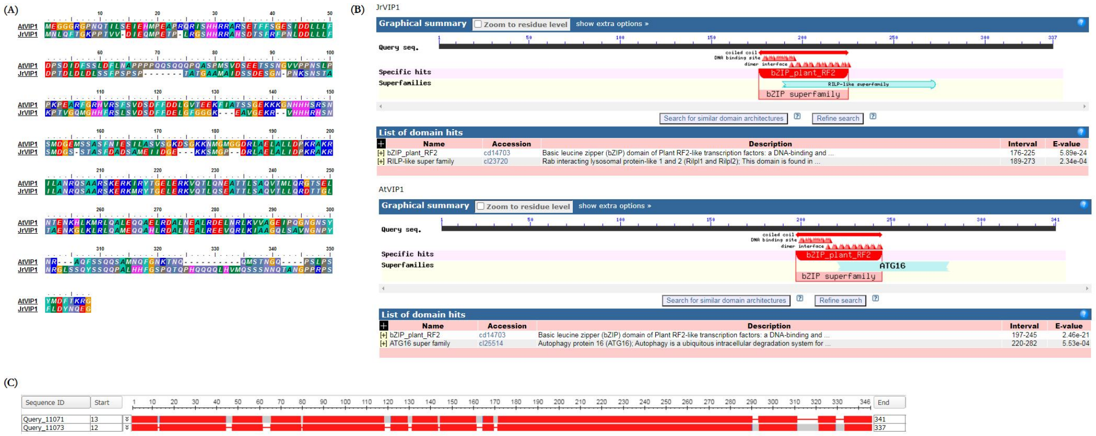

Fig. S3 Similarity and conservative domain analysis of JrVIP1. A, Alignment of JrVIP1 and AtVIP1. B, Similarity analysis using online blast in NCBI. C, The conservative domains existed in JrVIP1 and AtVIP1.
