# Supplementary material for: Walnut phosphatase 2A proteins interact with basic leucine zipper protein JrVIP1 to regulate osmotic stress response via calcium signaling
Source: For Res (Fayettev). 2024 May 6;4:e016. doi: 10.48130/forres-0024-0012 (PMC11543299; doi:10.48130/forres-0024-0012)
Supplement: Supplementary file 1 — Supplementary data to this article can be found online. [file forres-0024-0012-S1.zip › 10.48130_forres-0024-0012-Suppl-TableS1.pdf]

Table S1 The primers used for qRT-PCR analysis.

Gene names	Forward Primer	Reverse Primer
JrPP2A01	5'-CTGGAGGAAGTGATTCTG-3'	5'-GAACCTCACAGCATAGGT-3'
JrPP2A02	5'-GATTGACACAGACTGCAC-3'	5'-GGCGTGCTCTGTAAGAAT-3'
JrPP2A03	5'-GGAAGTTCTCTCAGGTCT-3'	5'-GACTCTGGAACTCAGTCT-3'
JrPP2A04	5'-AATGTGGTCGTGAACCAC-3'	5'-GACTTCAAGGTATCCGAG-3'
JrPP2A05	5'-TCCATGATCTTGCAGAGC-3'	5'-CGAAGGCACTCATCATAG-3'
JrPP2A06	5'-GATGAACCTCTTCGTGAG-3'	5'-CTCGACTTAGATCGGTAG-3'
JrPP2A07	5'-CGTCGAAGAGAGTAACGT-3'	5'-ATCCGATCTGGATACCTC-3'
JrPP2A08	5'-ACGCAAGTCCTCTAAGTC-3'	5'-AACCAGGCAAAGCCTCAT-3'
JrPP2A09	5'-GATCAGAAGCCTTGATGG-3'	5'-TGCATCTAGATCGTCAGC-3'
JrPP2A10	5'-GTCCTAATCGACGAGCTC-3'	5'-ATGGTGGAAGCAGCACAT-3'
JrPP2A11	5'-ACTGGGAACCACTTAGCT-3'	5'-AGCACCATTAGCTGTCTG-3'
JrPP2A12	5'-GCATACAGTAGCTCAAGG-3'	5'-GAGTCATGTAGTCTCGAC-3'
JrPP2A13	5'-GTCAGCATCTGTTGGAAG-3'	5'-GGATCGTGAAGACTTCAAG-3'
JrPP2A14	5'-ATCTGGACCGTCAGATTG-3'	5'-CTCAACTGAGTAGTAGCC-3'
JrPP2A15	5'-AGCAGTGACCTTCTGAGT-3'	5'-TCGTTGGATCGGTGAAGT-3'
