# Supplementary material for: Walnut phosphatase 2A proteins interact with basic leucine zipper protein JrVIP1 to regulate osmotic stress response via calcium signaling
Source: For Res (Fayettev). 2024 May 6;4:e016. doi: 10.48130/forres-0024-0012 (PMC11543299; doi:10.48130/forres-0024-0012)
Supplement: Supplementary file 1 — Supplementary data to this article can be found online. [file forres-0024-0012-S1.zip › 10.48130_forres-0024-0012-Suppl-TableS2.pdf]

Table S2 The primers used for construction of overexpression vectors and interaction related recombinants.

Recombinants	Forward Primer	Reverse Primer
35S::JrPP2A07	5’-ATCGTCTAGAATGTCAGACCTAGACAGGC-3’	5’-CGATGGTACCTCATAAAAAATAATCTGGT-3’
35S::JrPP2A09	5’-ATCGGGTACCATGTACAGTGGGTCCAGTGATG-3’	5’-CGATGAGCTCTCATTCTTCTTCTGGCTCTT-3’
35S::JrPP2A14	5’-ATCGGGATCCATGCCCTCGCACGCGGATCT-3’	5’-CGATGGTACCTTACAAAAAATAATCGGGAG-3’
AD-JrPP2A02	5’-TGGCCATTATGGCCCGGGATGATGTATGTATTGCTGTG-3’	5’-GACATGTTTTTCCCGGGTCAAAAAGGAGCTTCAAGTG-3’
AD-JrPP2A05	5’-TGGCCATTATGGCCCGGGATGGATTCGGTCCCTTCGAAC-3’	5’-GACATGTTTTTCCCGGGTTAAACCACCTGGCCAAAGGT-3’
AD-JrPP2A07	5’-TGGCCATTATGGCCCGGGATGTCAGACCTAGACAGGC-3’	5’-GACATGTTTTTCCCGGGTCATAAAAAATAATCTGGT-3’
AD-JrPP2A09	5’-TGGCCATTATGGCCCGGGATGTACAGTGGGTCCAGTGATG-3’	5’-GACATGTTTTTCCCGGGTCATTCTTCTTCTGGCTCTT-3’
AD-JrPP2A014	5’-TGGCCATTATGGCCCGGGATGCCCTCGCACGCGGATCT-3’	5’-GACATGTTTTTCCCGGGTTACAAAAAAATAATCGGGAG-3’
AD-JrVIP1	5’-TGGCCATTATGGCCCGGGATGAACCTGCAGTTCACCGG -3’	5’-GACATGTTTTTCCCGGGCTACCCCTCCTGGTTATAGT-3’
BD-JrPP2A02	5’-GGGAATTCCATATGATGATGTATGTATTGCTGTG-3’	5’-GCAGGTCGACGGATCCTCAAAAAGGAGCTTCAAGTG-3’
BD-JrPP2A05	5’-CATGGAGGCCGAATTCATGGATTCGGTCCCTTCGAAC-3’	5’-GCAGGTCGACGGATCCTTAAACCACCTGGCCAAAGGT-3’
BD-JrPP2A07	5’-CATGGAGGCCGAATTCATGTCAGACCTAGACAGGC-3’	5’-GCAGGTCGACGGATCCTCATAAAAAATAATCTGGT-3’
BD-JrPP2A09	5’-CATGGAGGCCGAATTCATGTACAGTGGGTCCAGTGATG-3’	5’-AAGGAAAAAAGCGGCCGCTCATTCTTCTTCTGGCTCTT-3
BD-JrPP2A14	5’-CATGGAGGCCGAATTCATGCCCTCGCACGCGGATCT-3’	5’-GCAGGTCGACGGATCCTTACAAAAAATAATCGGGAG-3’
BD-JrVIP1	5’-CATGGAGGCCGAATTCATGAACCTGCAGTTCACCGG-3’	5’-AAGGAAAAAAGCGGCCGCCTACCCCTCCTGGTTATAGT-3’
pET30a-JrPP2A07	5’-TAAGAAGGAGATATACATATGTCAGACCTAGACAGGCAAAT-3’	5’-GTGGTGGTGGTGGTGCTCGAGTAAAAAATAATCTGGTGCAGGTT-3’
pET30a-JrPP2A09	5’-TAAGAAGGAGATATACATATGTACAGTGGGTCCAGTGATG-3’	5’-GTGGTGGTGGTGGTGCTCGAGTTCTTCTTCTGGCTCTTCCTCA-3’
pET30a-JrPP2A14	5’-TAAGAAGGAGATATACATATGCCCTCGCACGCGGATCTGG-3’	5’-GTGGTGGTGGTGGTGCTCGAGCAAAAAATAATCGGGAGTCTTG-3’
pGEX-JrVIP1	5’-GATCTGAATTCGCGTGGATCCATGAACCTGCAGTTCACCGG-3’	5’-GTCACGATGCGGCCGCTCGACTACCCCTCCTGGTTATAGT-3’
