# Supplementary material for: Walnut phosphatase 2A proteins interact with basic leucine zipper protein JrVIP1 to regulate osmotic stress response via calcium signaling
Source: For Res (Fayettev). 2024 May 6;4:e016. doi: 10.48130/forres-0024-0012 (PMC11543299; doi:10.48130/forres-0024-0012)
Supplement: Supplementary file 1 — Supplementary data to this article can be found online. [file forres-0024-0012-S1.zip › 10.48130_forres-0024-0012-Suppl-TableS3.pdf]

Table S3 The *cis*-acting elements of 15 JrPP2A promoters.

Name	Function	Potential function
ABRE	Abscisic acid responsiveness	Hormone responsiveness
AAGAA-motif		
CCAAT-box	MYBHv1 binding site	
CGTCA-motif	MeJA-responsiveness	
GARE-motif	Gibberellin-responsive element	
P-box		
TATC-box		
TCA-element	Salicylic acid responsiveness	
TGACG-motif	MeJA-responsiveness	
TGA-element	Auxin-responsive element	
AAAC-motif	Light responsive element	Light responsiveness
ACA-motif	Part of gapA (in gapA-CMA1)	
ACE	Light responsiveness	
AE-box	Part of a module for light response	
AT1-motif	Part of a light responsive module	
ATC-motif	Part of a conserved DNA module involved in light responsiveness	
ATCT-motif		
Box 4		
chs-CMA1a	Part of a light responsive element	
chs-CMA2a		
chs-CMA2a		
GA-motif		
Gap-box		
GATA-motif		
GATT-motif		
G-box	*Cis*-acting regulatory element involved in light responsiveness	
GT1-motif	Light responsive element	
I-box	Part of a light responsive element	
MRE	MYB binding site involved in light responsiveness	
Sp1	Light responsive element	
TCCC-motif	Part of a light responsive element	
TCT-motif		
WRE3	Wound-responsive element	Abiotic response
ARE	Anaerobic induction	
DRE1	Drought response	
GC-motif	Enhancer-like element involved in anoxic specific inducibility	
LTR	Low-temperature responsiveness	
MBS	MYB binding site involved in drought-inducibility	
MYB	Drought-inducibility	
MYB recognition site	Dehydration stress response	
Myb-binding site	Stress response	
MYB-like sequence		
MYC	Drought and abscisic acid responsiveness	
STRE	Heat stress response	

TC-rich repeats	Defense and stress responsiveness	
W box	Dehydration responsiveness	
WUN-motif	Wound-responsive element	
A-box	*Cis*-acting regulatory element	Plant growth and devlopment
AT-rich sequence	For maximal elicitor-mediated activation (2copies)	
CAT-box	Related to meristem expression	
circadian	Involved in circadian control	
GCN4_motif	Involved in endosperm expression	
MBSI	MYB binding site involved in flavonoid biosynthetic genes regulation	
O2-site	Involved in zein metabolism regulation	
RY-element	Involved in seed-specific regulation	
