# Supplementary material for: Walnut phosphatase 2A proteins interact with basic leucine zipper protein JrVIP1 to regulate osmotic stress response via calcium signaling
Source: For Res (Fayettev). 2024 May 6;4:e016. doi: 10.48130/forres-0024-0012 (PMC11543299; doi:10.48130/forres-0024-0012)
Supplement: Supplementary file 1 — Supplementary data to this article can be found online. [file forres-0024-0012-S1.zip › 10.48130_forres-0024-0012-Suppl-TableS4.pdf]

Table S4 Distribution of the number of *cis*-acting elements of 15 JrPP2As

**Gene Name**	**Hormone responsiveness**	**Light responsiveness**	**Abiotic response**	**Plant growth and development**
JrPP2A01	8	7	20	2
JrPP2A02	2	5	14	7
JrPP2A03	6	10	16	1
JrPP2A04	9	13	7	3
JrPP2A05	14	6	12	2
JrPP2A06	6	21	8	1
JrPP2A07	3	6	15	2
JrPP2A08	7	11	21	5
JrPP2A09	4	10	14	2
JrPP2A10	8	12	30	2
JrPP2A11	9	14	12	4
JrPP2A12	9	14	13	0
JrPP2A13	7	16	16	0
JrPP2A14	10	11	11	1
JrPP2A15	7	9	10	2
