# Supplementary material for: Walnut phosphatase 2A proteins interact with basic leucine zipper protein JrVIP1 to regulate osmotic stress response via calcium signaling
Source: For Res (Fayettev). 2024 May 6;4:e016. doi: 10.48130/forres-0024-0012 (PMC11543299; doi:10.48130/forres-0024-0012)
Supplement: Supplementary file 1 — Supplementary data to this article can be found online. [file forres-0024-0012-S1.zip › 10.48130_forres-0024-0012-Suppl-TableS5.pdf]

Table S5 The number of cis-elements in the JrPP2A promoters

Gene Name	ABRE	CCAAT	CGTCA	GARE	P-box	TATC	TCA-element	TGACG	TGA-element	AAGAA
JrPP2A01	2	1	1	1	1					2
JrPP2A02										2
JrPP2A03	1	1	1		1			2		
JrPP2A04	4	1	1			1	2			
JrPP2A05	6	1	1		1		2			3
JrPP2A06		2	2							2
JrPP2A07							1		1	1
JrPP2A08	1	1	1			1	1			2
JrPP2A09	4									
JrPP2A10	3	1	1	1					1	1
JrPP2A11	4	1	1						3	
JrPP2A12	4	1	1						3	
JrPP2A13	3	1	1						2	
JrPP2A14	3	1	1				1			4
JrPP2A15		2	2				1		1	1
sum	35	14	14	2	3	2	8	2	11	18
Gene Name	AAAC	ACA	ACE	AE-box	AT1	ATC	ATCT	Box 4	chs-CMA1a	chs-CMA2a
JrPP2A01							1	2		1
JrPP2A02					1			2		
JrPP2A03				1				3		1
JrPP2A04						1	1	1	2	
JrPP2A05				1				1		
JrPP2A06					1			7		
JrPP2A07										
JrPP2A08				1				2		1
JrPP2A09										
JrPP2A10		1						1		
JrPP2A11				1				4		
JrPP2A12				1				4		
JrPP2A13			2		1			5	2	
JrPP2A14								2		
JrPP2A15	1			1			1			
sum	1	1	2	6	3	1	3	34	4	3
Gene Name	GA-motif	Gap-box	GATA	GATT	G-box	GT1	I-box	MRE	Sp1	TCCC
JrPP2A01			1		2					
JrPP2A02					1	1				
JrPP2A03			1		2			1		
JrPP2A04	1			1	4			1		
JrPP2A05					3					1
JrPP2A06			1		9	2				
JrPP2A07					4				1	1
JrPP2A08			1		2	1		1		1
JrPP2A09					2	2		3	1	2
JrPP2A10					5			3		1
JrPP2A11	1				5		1			

JrPP2A12	1				5		1			
JrPP2A13			1					2	1	2
JrPP2A14		1	2		4	1		1		
JrPP2A15						2				2
sum	3	1	7	1	48	9	2	12	3	10
Gene Name	TCT	WRE3	ARE	DRE1	GC-motif	LTR	MBS	MYB	MYB recognition	Myb-binding
JrPP2A01		1	1					6		1
JrPP2A02		2	3			2		1		
JrPP2A03	1		3			2	3	5		
JrPP2A04	1	1				1			1	
JrPP2A05		2	4			1		2		
JrPP2A06	1		2					1		
JrPP2A07			2		1		2	2		
JrPP2A08	1		6	1		1	1	6		2
JrPP2A09						1		5		
JrPP2A10	1	2	3			3		4		2
JrPP2A11	2		2				1	2		
JrPP2A12	2		2				1	2		
JrPP2A13			4					3		
JrPP2A14		1	1			1	1	3		
JrPP2A15	2	1	2				1	5		
sum	11	10	35	1	1	12	10	47	1	5
Gene Name	MYB-like	MYC	STRE	TC-rich repeats	W box	WUN	A-box	AT-rich	CAT-box	circadian
JrPP2A01	1	4	5	1						
JrPP2A02	1	3			1	1				
JrPP2A03		1		1		1		1		
JrPP2A04		2	1	1			1		1	
JrPP2A05		3								
JrPP2A06		4	1							
JrPP2A07		3	2	2		1				1
JrPP2A08	2		2				1		2	
JrPP2A09	1	2	3		2					1
JrPP2A10		4	8	1		3	2			
JrPP2A11	1	2	1	3						
JrPP2A12	1	2	1	3	1					
JrPP2A13	1	4	4							
JrPP2A14		3			1		1			
JrPP2A15	1							1		1
sum	9	37	28	12	5	6	5	2	3	3
Gene Name	GCN4_motif	MBSI	O2-site	RY-element						
JrPP2A01			2							
JrPP2A02			7							
JrPP2A03										
JrPP2A04			1							
JrPP2A05	1		1							
JrPP2A06				1						
