# Supplementary material for: Walnut phosphatase 2A proteins interact with basic leucine zipper protein JrVIP1 to regulate osmotic stress response via calcium signaling
Source: For Res (Fayettev). 2024 May 6;4:e016. doi: 10.48130/forres-0024-0012 (PMC11543299; doi:10.48130/forres-0024-0012)
Supplement: Supplementary file 1 — Supplementary data to this article can be found online. [file forres-0024-0012-S1.zip › 10.48130_forres-0024-0012-Suppl-FigureS1.pdf]

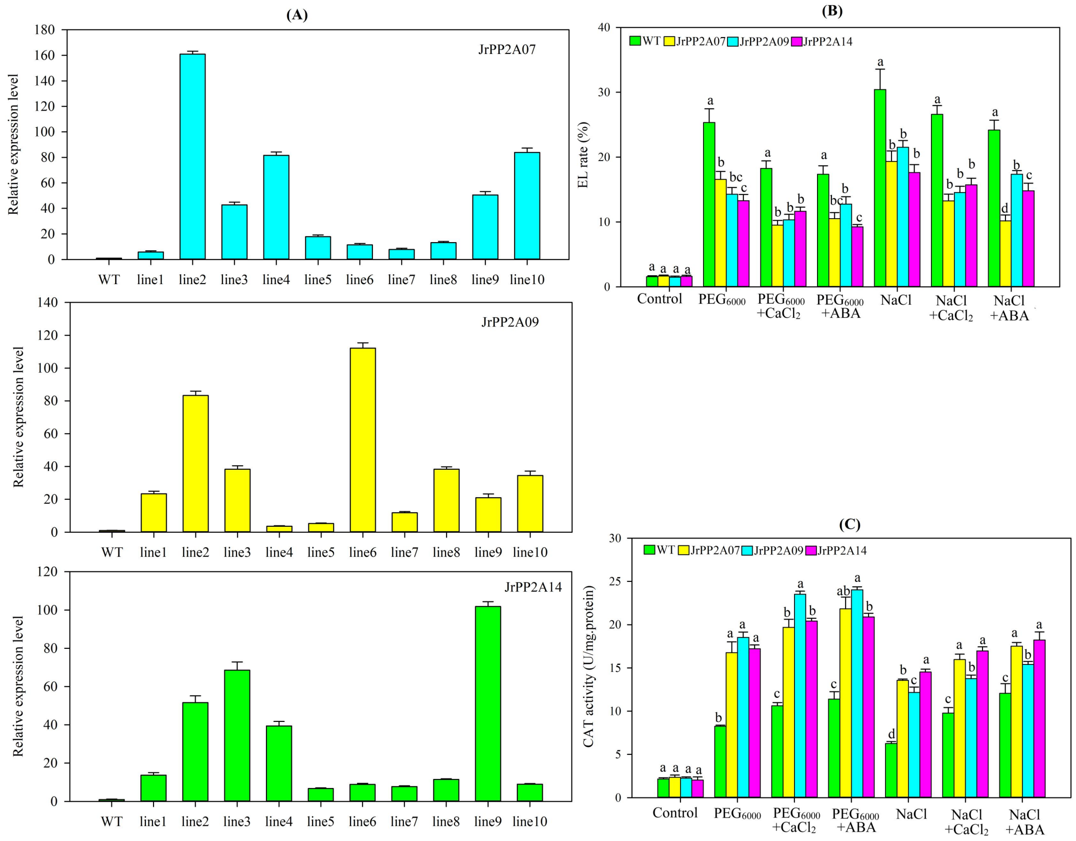

Fig. S1 The expression, EL rate and CAT activity of *JrPP2A07*, *JrPP2A09*, *JrPP2A14* transgenic lines. A, The expression level of *JrPP2A07*, *JrPP2A09*, *JrPP2A14* in transgenic lines. B-C, the EL rate and CAT activity under the treatments of PEG_{6000}, NaCl, $PEG_{6000}+CaCl_2$, PEG_{6000}+ABA, NaCl, NaCl+ $CaCl_2$, NaCl+ ABA for 3 days, which was consistent with figure 5. The significant differences among WT, *JrPP2A07*, *JrPP2A09* and *JrPP2A14* were marked with lowercase ($P<0.05$).
